# Area Selective Atomic Layer Deposition for the Use on Active Implants: An Overview of Available Process Technology

**DOI:** 10.1002/adhm.202403149

**Published:** 2024-12-26

**Authors:** Nicolai Simon, Thomas Stieglitz, Volker Bucher

**Affiliations:** ^1^ Institute for MicroSystems Technology (iMST) Faculty of Mechanical & Medical Engineering, Furtwangen University D‐78120 Furtwangen im Schwarzwald Germany; ^2^ Laboratory for Biomedical Microtechnology Dept. Microsystems Eng.‐IMTEK University of Freiburg D‐79110 Freiburg Germany; ^3^ BrainLinks‐BrainTools // IMBIT University of Freiburg D‐79110 Freiburg Germany; ^4^ Bernstein Center Freiburg University of Freiburg D‐79110 Freiburg Germany

**Keywords:** active implantable medical devices, area selective atomic layer deposition, coating technologies, encapsulation strategies, surface reactions

## Abstract

Area‐selective atomic layer deposition (ASD) is a bottom‐up process that is of particular importance in the semiconductor industry, as it prevents edge defects and avoids cost‐intensive lithography steps. This approach not only offers immense potential for the manufacture of active implants but can also be used to improve them. This review paper presents various processes that can be used for this purpose. It also identifies aspects that shall be considered when implementing such a process for medical applications. For example, the inherent selectivity can be used to produce new biosensors, the passivated ASD can be used to encapsulate polymer‐based implants, and the activated ASD can be used to improve electrode performance. Finally, the aspects that shall be considered in a coating for active implants are highlighted. ASD therefore offers great potential for use on active implants.

## Graphical abstract

Area‐selective atomic layer deposition (ASD) offers wide‐ranging potential for the use on active implants. As the active implants consist of heterogeneous materials, this provides an excellent basis for the application of ASD. In this review, processes for the application on active implants and possible application scenarios are highlighted.

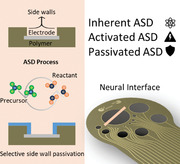

## 1 Introduction

Restoring hearing,^[^ ^1^ ^]^ sight,^[^ ^2^ ^]^ or movement^[^ ^3^ ^]^ is no longer a vision of the future with the use of suitable active implantable medical devices (AIMDs). These AIMDs, which interact with the nervous system of the human body are called neural implants. They interface directly to the brain, the spinal cord (i.e., the central nervous system) or to the peripheral nervous system^[^ ^4^ ^]^ to modulate activity during intervention, to restore lost body functions or to control prosthetic devices.^[^ ^5^ ^]^ Neural implants are not only used for stimulation purposes but also for recording bioelectrical activity. This neural activity can be directly recorded by electrodes, as change of the magnetic field resulting from the change of the electrical current and via fluorescence by imaging ion changes via fluorescent markers.^[^ ^6^, ^7^ ^]^ For stimulation, a charge injection, initiated by the implant, depolarizes the surrounding tissue and thus triggers a stimulus.^[^ ^8^ ^]^ Recording and stimulation electrodes have to be made from materials exhibiting low noise and delivering sufficient amount of charge without corrosion. Electrode materials with these electrochemical properties include platinum,^[^ ^9^ ^]^ gold,^[^ ^10^ ^]^ tungsten,^[^ ^11^ ^]^stainless steel,^[^ ^12^ ^]^ iridium (oxide),^[^ ^13^ ^]^ carbon nanotubes,^[^ ^14^ ^]^ and conductive polymers.^[^ ^15^ ^]^ The interface between the electrode and the tissue should be stable over time to get reliable functionality. As all components of a neural implant are in direct contact with human tissue, they are exposed to various stressors. Ions, water and mechanisms of the foreign body reactions like change in pH can impair the function of the implant, which eventually lead to total device failure. Furthermore, these implants must not harm the human body and biological tissue through toxic substance excretions, dangerous currents, or inflammatory reactions.

Electrodes and sometimes the entire implant are embedded in flexible polymers as substrate and insulation layer. However, polymers do not offer sufficient protection of electronic components against body fluids since they are not hermetic and do not sufficiently prevent water vapor transition. To overcome these challenges, a flexible hermetic encapsulation is being sought that significantly increases the durability, longevity, and biological compatibility of the implant by limiting the water vapor transition rate and delivering hermeticity.

Atomic layer deposition (ALD) processes deliver ultra‐thin ceramic barrier coatings and promise to meet these challenges.^[^ ^16^ ^]^ This coating technology allows the deposition of highly conformal, defect‐free layers with a thickness control in the Ångstrom (10^−10^ m) level. These layers are called nanolaminates or nanomembranes. In ALD, the layer‐forming chemicals, the so‐called precursors and reactants, are introduced into the reaction chamber in clearly separated process steps. The precursors react with the surface in a self‐limiting manner such that no further precursor can react. The reactant completes the reaction and makes the surface reactive again.^[^ ^17^ ^]^ With subsequent coating cycles, layer‐by‐layer growth can be achieved. Due to the cyclic reaction chemistry of this process, lateral control of the growth is challenging. The applied coating conformally coats the entire implant, which is favorable, but comes with drawbacks. Due to the dielectric properties of the layer, it deteriorates the recording and sensing performance of the electrodes. To take advantage of the ALD coating, while not limiting electrode performance, the process must be modified to coat only the desired surfaces.

This technique is called area selective atomic layer deposition (ASD) and it already plays an important role for the production of future devices^[^ ^18^ ^]^ and in semiconductor industries, since it meets the requirement to produce smallest features due to its self‐aligned fabrication schemes.^[^ ^19^ ^]^ Instead of performing lithography, lift‐off and etching steps for each layer within a device stack, certain layers can be deposited in a bottom‐up approach using ASD, thus eliminating the edge placement errors and saving cost and time.^[^ ^20^ ^]^ ASD can be used to produce high performance catalysts,^[^ ^21^ ^]^ by producing core‐shell nanoparticles^[^ ^22^ ^]^ and nanoelectronics applications.^[^ ^23^ ^]^ Due to these advantages, ASD offers a promising approach in the production, encapsulation and improvement of active implants.

Methods for ASD are reviewed in this paper presenting and discussing aspects of process control parameters, selection of suitable chemicals and exploiting surface chemistry of heterogeneity regions of active implants.

## 2 Active Implantable Medical Devices

Active implantable medical devices (AIMDs) are used to replace and restore lost functions of the human organism,^[^ ^24^ ^]^ monitor body functions,^[^ ^25^ ^]^ and interact with them.^[^ ^26^ ^]^ AIMDs need either sensor or actuator systems to be able to perceive the environment or interact with it, respectively. An energy source needs to power such an implant and electronic circuitry controls all actions. As implants, these systems are either fully or partially inserted into the human body and remain there after the procedure of implantation.^[^ ^27^ ^]^

Applications include recording and monitoring of bio signals and their pathophysiological changes in the ear,^[^ ^28^ ^]^ the brain,^[^ ^29^ ^]^ and muscles,^[^ ^30^ ^]^ for example. The most widespread AIMD is the cardiac pacemaker to restore the rhythm of the heart by electrical stimulation. It consists of a hermetically sealed titanium case that protects the built‐in electronics and a battery. The electrodes are connected via cables to the housing and transduce the excitation signal into the tissue.

**Figure 1:**
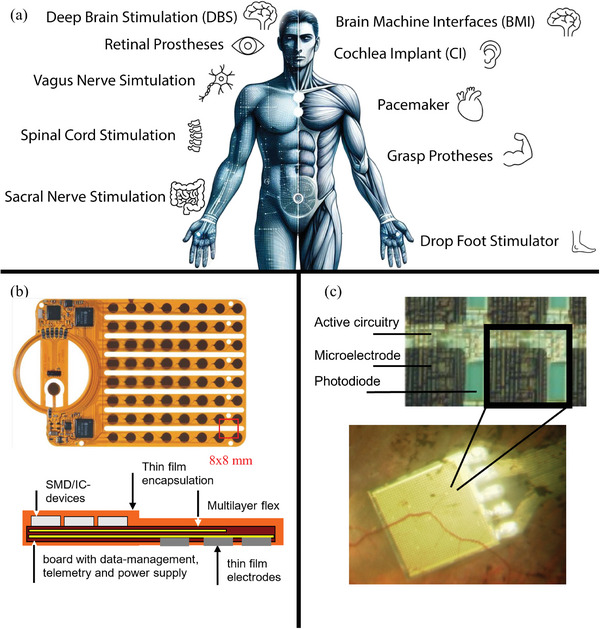
a) Examples of neural implants. Deep brain stimulation is used for the therapy of Parkinson's disease and essential tremor, brain‐machine interfaces are used for the control of external devices, the cochlear implant as well as retinal prostheses are used for the restoration of hearing and vision, respectively. Vagus nerve stimulation is used for the treatment of epilepsy, the pacemaker for the treatment of cardiac arrhythmia, and spinal cord stimulation for the treatment of chronic pain. The grasp prosthesis is used for gripping movement and sacral nerve stimulation is used to treat incontinence. The drop foot stimulator restores the gait pattern. b) Example of the “Brainboard”, a flexible implant to record activities in the brain. Reproduced with permission from NMI Reutlingen, Germany. c) Example of a Chip for subretinal electrical stimulation manufactured by the Retina Implant AG.

Neural implants (**Figure** **1**a) are a growing subgroup of AIMDs. They interact with the nervous system of the human body via electrodes which are tailored to the target tissue and application.^[^ ^31^ ^]^ Examples include treatment of Parkinson's disease,^[^ ^32^ ^]^ chronic intractable pain,^[^ ^33^ ^]^ and incontinence.^[^ ^34^ ^]^

While most clinical implants use precision mechanics electrodes connected to hermetic titanium packages, new developments rely on microsystems technologies using either silicon or polymers as substrate and insulation material, distributed electronics and thin‐films to deliver protection of electronics.^[^ ^6^ ^]^ An example of a polymer‐based implant is shown in Figure 1b. The “Brainboard” was developed for stroke patients who can no longer grasp due to neuromuscular damage. It should record the patient's will to move and electrically stimulate the paralyzed forearm via a control system and thus restore motor function. The entire electronics are encapsulated in a flexible polymer. Stimuli are recorded and sent to the brain tissue via electrodes. A silicon‐based implant is shown in Figure 1c. The retinal implant is intended to be used to replace degenerated photoreceptor cells. An electrical signal is generated via photodiodes when light is applied. The signal is detected by the intact nerve cells in the eye and sent to the brain, where a visual impression is created.^[^ ^35^ ^]^

Limitation in space, conformability at the material‐tissue interface and requirements on system complexity drive technological developments like chip‐film patch (CFP) techniques.^[^ ^36^ ^]^ There, a silicon chip is thinned down to a few tens of micrometers and embedded between two polyimide films. The electrodes are connected to the silicon chip via conductor tracks and are in direct contact with the tissue on the other side. The thickness of this CFP system is less than 70 µm and can be used between the surface of the brain and the skull bone. All needed electronics, traces and electrodes are sandwiched between the two polymeric films. While planar surfaces are easy to coat, sidewalls of openings for electrodes are entries for water and ions which cause adhesion loss and failure at the polymer‐metal interface. Selective coating techniques are the starting point for our consideration of area selective ALD coating processes.

### 2.1 Strategy for Sidewall Passivation

**Figure 2:**
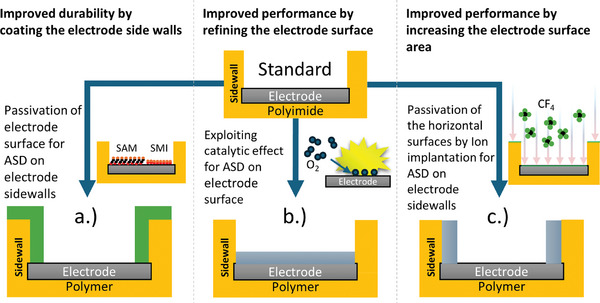
Schematic cross section of a neurotechnical interface with possible ASD processes. a) Sidewall passivation of the electrodes using self‐assembled monolayer (SAM) or small molecule inhibitor (SMI). By using these molecules, the layer growth on the electrode surface is prevented so that a layer is only deposited on the polymer (e.g. polyimide). b) Utilization of the catalytic effect of certain noble metals as electrode material. Platinum dissociates oxygen on the surface, creating an active surface that allows selective coating. c) Enlargement of the electrode surface by topographically selective ALD. Using ion implantation, the horizontal surface is passivated by fluorine compounds so that ALD growth can only take place on vertical surfaces.

The side walls of the electrode openings in active implants provide a surface of attack for penetrating body fluid and are thus a significant weak point. Due to the nature of the process, the electrode opening is performed using a plasma etching process, which roughens the side walls. If the implants are to be encapsulated with a coating process, these areas are of challenge. Electrode openings have a high aspect ratio, so a highly conformal encapsulation method is required. Accordingly, a complex masking process is also necessary to be able to produce such a sidewall passivation in the passivation process.^[^ ^37^ ^]^ However, using ASD by selecting suitable precursors, pre‐treatment methods and process control, this passivation should be possible without complex and time‐consuming lithography, by just deactivating layer growth on the electrode surface using suitable inhibitors. (**Figure** **2**a).

### 2.2 Strategy for Improved Electrode Performance

The impedance of the electrode surface determines the signal‐to‐noise ratio (SNR) of recorded bioelectrical signals.^[^ ^38^ ^]^ The impedance depends on the electrode material and surface area, the type of electrolyte in contact and the temperature, while the latter two parameters are given by the environment in the body. The impedance decreases with a larger (electrochemical) contact surface. The surface modification of the electrode and the associated impedance reduction should be fully integrated into an ASD process to leave established manufacturing processes of neural implants unchanged. To this end, ASD will be used to coat existing electrode surfaces with an impedance‐reducing layer (Figure 2b).

### 2.3 Strategy for Increasing the Geometric Surface of the Electrode

Bioelectrical signals are stimulated and recorded via the electrode surfaces of the neural implant. During stimulation, all processes need to stay in the reversible range to maintain electrode longevity. Chemical safe charge injection limits must not be exceeded.^[^ ^39^ ^]^

Considering electrodes that are embedded in a polymer substrate, the electrode surface can be extended to the side walls with a suitable selection of area selective ALD. Thus, not only the 2d surface contributes to charge injection, but the electrode becomes 3D via the coating of the side walls (Figure 2c). Since reactive ion etching is the standard technology to open the electrodes, the side walls have a high roughness and therefore a large geometric surface, which can be coated with a suitable electrode material in a highly conformal manner by ASD. This would largely increase the electrochemical surface area without changing the geometric footprint area of the electrode.

## 3 Area Selective Atomic Layer Deposition for the Use on Active Implants

Neural implants need to withstand the harsh environments in the body over their complete lifetime. Therefore, they must be coated with a suitable protective layer. It must be hermetic, biocompatible, and thin to maintain flexibility. Conventional encapsulation strategies such as Parylene‐C and Polyimide can protect the implant with internal electronics for several days but fall short in the case of a long‐term implant since polymers are not hermetic. Atomic layer deposition (ALD) is a modified form of chemical vapor deposition (CVD) and achieves highly conformal inorganic coatings with thin, defect‐free layers that is ideally suited to produce barrier coatings. Thereby ALD reduces the water vapor transmission rate (WVTR) and brings the system toward hermetic encapsulation. The process ALD was developed independently in the 1960s under the name “molecular layering” (ML) in the Soviet Union, and in the mid‐1970s under the name atomic layer epitaxy (ALE) in Finland.^[^ ^40^ ^]^

**Figure 3:**
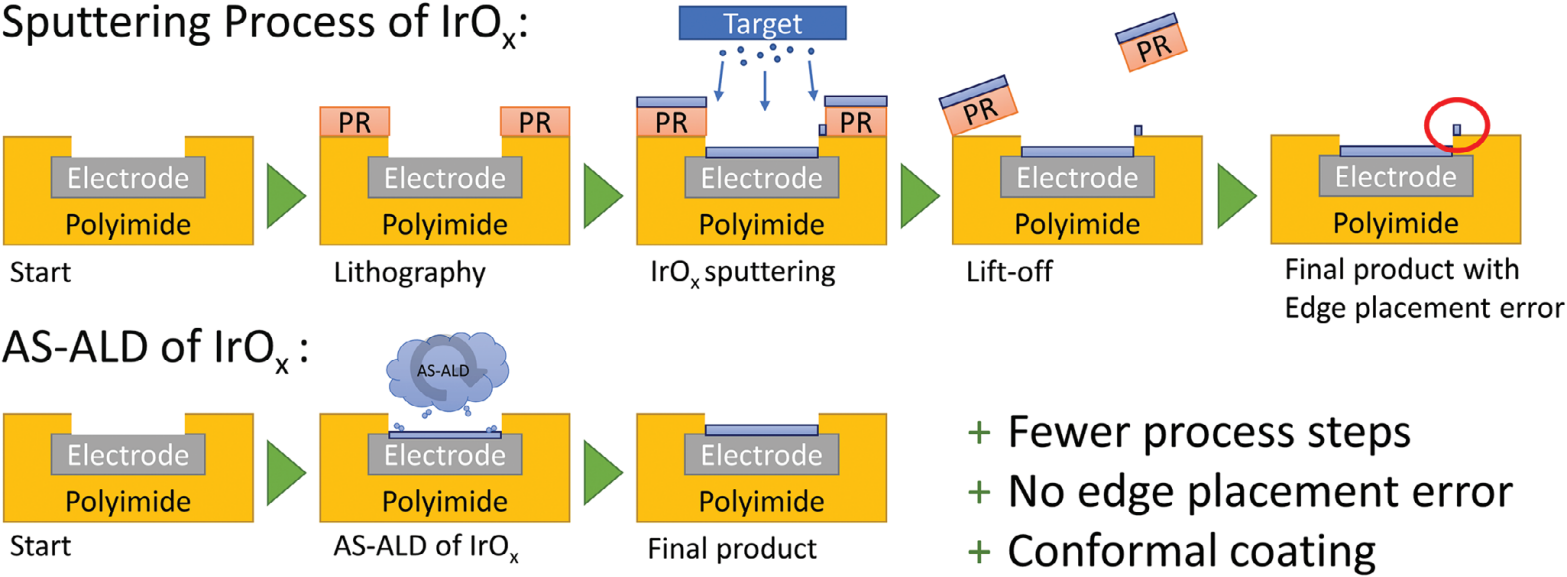
Advantages of area selective atomic layer deposition compared to conventional sputter coating processes for the production of high‐performance electrodes using IrO_x_.

Atomic layer deposition has been used for coating medical devices and biological materials to improve their performance or realize novel manufacturing techniques^[^ ^41^ ^]^ and for the use of biosensors.^[^ ^42^ ^]^ It can improve biocompatibility,^[^ ^43^ ^]^ increase corrosion resistance,^[^ ^44^ ^]^ achieve better functionality,^[^ ^45^ ^]^ and provide decreased cytotoxicity.^[^ ^46^ ^]^ Various encapsulation strategies are used to generate a barrier layer around the implant that prevents the intrusion of electrolyte‐containing body fluid and the leakage of potential cytotoxic substances of the implant. Xie et al.^[^ ^47^ ^]^ and Minnikanti et al.^[^ ^48^ ^]^ showed a strong improvement of the barrier property by studying a bilayer system of aluminum oxides deposited by ALD and Parylene‐C. Westerhausen et al.^[^ ^49^ ^]^ investigated the barrier effect of a multilayer coating consisting of aluminum oxide and titanium oxide with accelerated aging. The degradation rate of the layers decreased with increasing layer thickness. Nanostructured platinum grass leads to a reduction in impedance by increasing the surface area of electrodes.^[^ ^50^ ^]^ If this structure would be covered via sputtered iridium oxide films (SIROF), the surface would be smoothed, and the effect of a big specific surface would be weakened. ALD coatings could leave this surface virtually unchanged in its morphology, combining the advantages of nano grass with the advantages of ALD coated iridium oxide,^[^ ^51^ ^]^ eventually resulting in a high‐performance electrode. In **Figure** **3** the advantage of an ASD process compared to a conventional sputter coating process is shown. This illustration shows a schematic cross section of an electrode. This example shows that an ASD can save process steps, increase process reliability and improve the quality of the coatings.

The quintessence of ALD is the self‐limiting growth of the layers. In conventional CVD, the precursors, are introduced into the reactor and then react with the surface to form the layer under the supply of energy. ALD is dependent on several parameters which need to be in a dynamic equilibrium to allow reproducible growth in the so‐called ALD‐window. The precursors and reactants are introduced into the reactor in clearly separated process steps, where they react on the surface in a self‐limiting manner. In the first step, a precursor is introduced into the reactor, whereas self‐limiting surface reaction takes place. The excess precursor is purged away in a second step. Now the reactant is used, which enables renewed growth by generating new active sites on the previously reacted precursor layer. After a further purging step, the process cycle starts again. Thus, ALD growth is cycle dependent.^[^ ^52^ ^]^

Layers produced by ALD are highly conformal and the coating thickness can be controlled at the Ångstrom level. For growth to occur, the precursor needs species to which it can bind. These species are called active sites and describe centers on the surface with high energy like functional groups, defects, or imperfections on the substrate surface. In the best case, chemically active groups, like hydroxyl groups are assumed. If there are many active sites on a surface, not all of them can be occupied, as already adsorbed precursor blocks surrounding free sites via steric hindrance which limits the saturation of the surface. The less active sites are present, the faster saturation is reached.^[^ ^53^ ^]^ The temperature at which the saturation of the surface is self‐limiting is called the ALD window. In this window, the layer growth per cycle (GPC) is almost constant.^[^ ^52^ ^]^ Higher temperatures within the window can lead to a slight change in the GPC, as additional reactions or desorption can occur without the loss of the self‐limiting character.^[^ ^54^ ^]^

**Figure 4:**
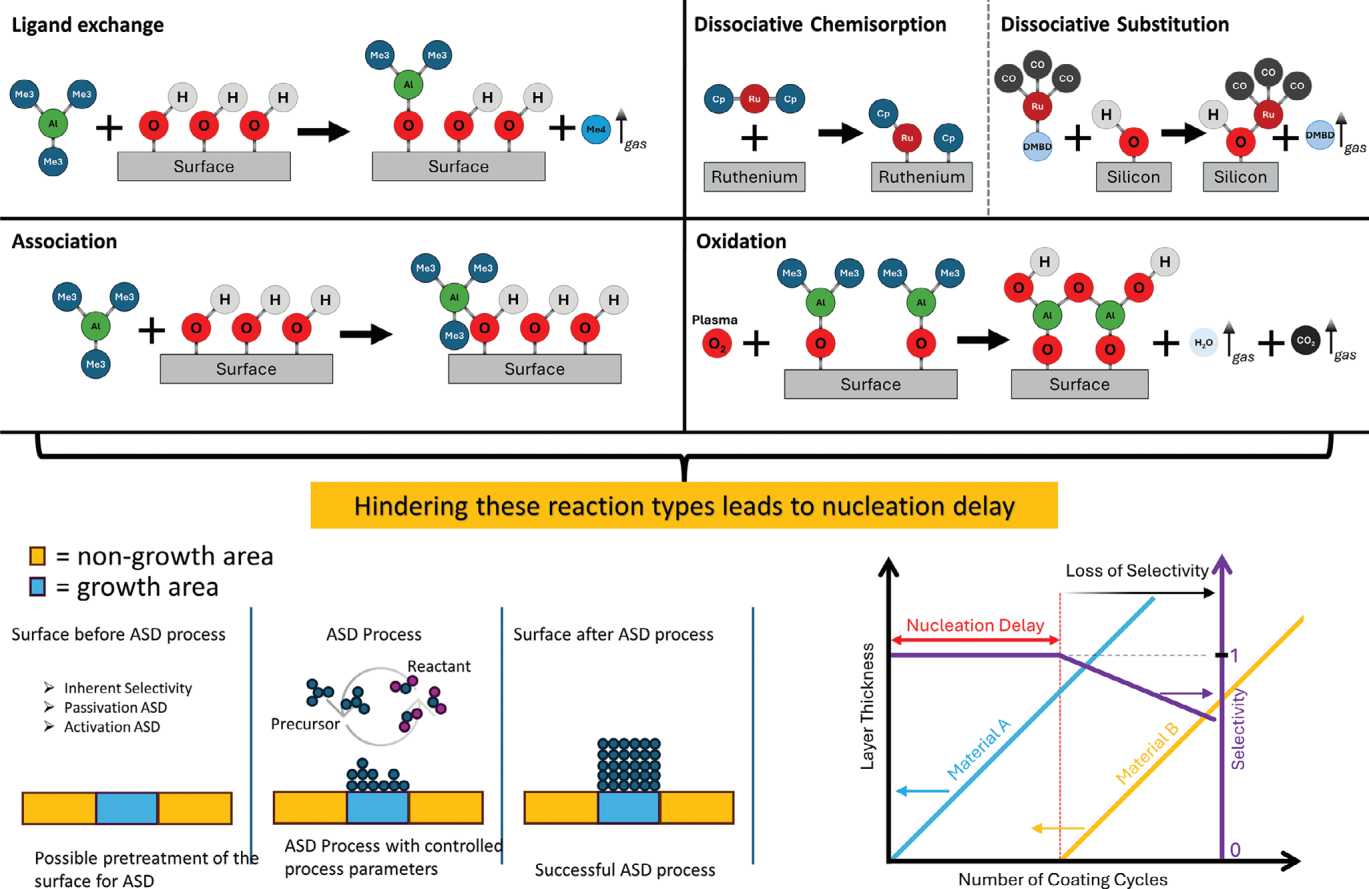
Schematic representation of the reaction mechanisms during the ALD process and fundamental reaction for achieving the area‐selective atomic layer deposition process.

The growth of the layer begins with the adsorption of the precursor at the active sites. Physisorption occurs first, whereby no bonds are broken, or by‐products generated and can therefore be assumed to be reversible. If the reaction kinetics allow it, the precursor can react once or even several times, breaking bonds and forming a covalent bond with the surface. This chemisorption is irreversible and can take place in several ways. The five most typical reaction mechanisms are described below (**Figure** **4**, upper part).

In the ligand exchange reaction, a ligand of the precursor is exchanged with a species of active surface group. These reactions take place, for example, with organometallic precursors such as trimethylaluminum (TMA) and a hydroxyl group on the surface. The ligand of the precursor acts as a Brønsted base and removes the proton from the hydroxyl group, which acts as a Brønsted acid. They form volatile methane, which can be purged away and the aluminum undergoes covalent bonding with the oxygen of the surface group.^[^ ^55^ ^]^

Chemisorption can also take place via a dissociation reaction. During dissociation, the bonds of a molecule are broken under the influence of energy. This type of reaction occurs on surfaces with high energy, as found in noble metals such as platinum or ruthenium. Oxygen molecules that are used in the reactant step can dissociate atomically on the substrate surface and thus increase their reactivity.^[^ ^56^ ^]^ The dissociative chemisorption (Figure 4, upper part) of the precursor bis(cyclopentadienyl)ruthenium(II) (Ru(Cp)_2_) directly bonds on a high energy surface like ruthenium^[^ ^57^ ^]^ or takes place as dissociative substitution reaction of 2,3‐Dimethylbutadiene ruthenium tricarbonyl (Ru(DMBD(CO)_3_) on a hydroxyl group on a silicon surface.^[^ ^58^ ^]^

The association reaction does not break any bonds but can be regarded as a self‐limiting reaction due to its binding character. The precursor TMA comes as a dimer. At higher temperatures, the dimer dissociates into a monomer and thus makes the aluminum atom accessible for an association reaction with a hydroxyl group, in which the oxygen atom can contribute two electrons for bonding to the 3p orbital of the aluminum. Although no bonds are broken, the precursor occupies the active site and contributes to the saturation of the surface. In the case of processes with halide precursors, it is assumed that some of these precursors associate with the surface.^[^ ^55^ ^]^

Oxidation reactions occur mainly in the reactant step, in which the remaining ligands of the precursor are combusted using a strong oxidizing agent such as oxygen, ozone or oxygen plasma to create new active sites.^[^ ^59^ ^]^ In the case of ALD of noble metals, where the oxygen is dissociated at the surface, it can oxidize the ligands in the subsequent precursor step.^[^ ^60^ ^]^ More detailed information about the surface reactions can be found in the work of Richey et al.^[^ ^61^ ^]^

The precursors used in the ALD react with preferred reaction kinetics. For example, precursors with a cyclopentadienyl ligand (Cp) are resistant to a ligand exchange reaction. With this precursor, the aromatic ring must be broken, so that HCp can be formed. This requires high‐energy metal surfaces, which dissociate this precursor. On the other hand, there are precursors that bind to the surface via an acid/base reaction, which require a hydroxyl‐terminated surface. If the preferred reaction kinetics are not present on the surface, a nucleation delay occurs. This is the period until a constant layer growth, or sufficient nucleation sides have been formed. However, a precursor can chemisorb on a surface contrary to its preferred reaction kinetics, but this can take several hundred coating cycles until growth is initiated either on defects on the substrate surface or via pyrolysis of the precursor. If a substrate is made off different chemical surfaces, this property can be exploited to perform area selective atomic layer deposition (ASD). In implant technology, where a wide variety of materials such as platinum, gold or iridium are often used as electrode materials and polyimide, Parylene‐C or polydimethylsiloxane are used as substrate materials, it offers immense potential for the use of the ASD process to increase the performance of said.

### 3.1 Area Selective ALD

Area selective atomic layer deposition is based on the exploitation of the nucleation delay. It describes the period before a significant number of nucleation sites have been formed, or a stable GPC is reached. It occurs due to lack of chemisorption of the precursor on the substrate. This is the case when the preferred reaction mechanism of the precursor is incompatible with the surface. Thus, growth is mainly initiated at defect sites or by pyrolysis of the precursor to form nucleation clusters, which is also the main cause for failing an ASD process. When enough clusters have been formed and stable growth per cycle occurs, an ALD process takes place.

In an ASD process, a distinction is made between two surfaces. The first, described as the growth area on which ALD growth is desired. On the non‐growth area, the growth should be prevented. (Figure 4, lower part). Quantifying an ASD is done using the **Equation** (**1**) of Gladfelter et al.^[^ ^62^ ^]^

(1) $$\mathrm{Selectivity}=\frac{\Theta_{\mathrm{GA}}-\Theta_{\mathrm{NGA}}}{\Theta_{\mathrm{GA}}+\Theta_{\mathrm{NGA}}}$$

*Θ*_GA_ and *Θ*_NGA_ represents the amount of material after the ASD process on the growth area and non‐growth area, respectively.

ASD is categorized into inherent, activated, and passivated deposition.^[^ ^63^ ^]^ The inherent selectivity describes the selectivity given by “nature”, due to the chemical character of the surface which leads to a nucleation delay. Activated ASD is achieved by using a patterned flux of high energy sources, like photons, electrons, or ions. In addition, exploiting the catalytic behavior of some materials can be considered as activated ASD. Passivated ASD is carried out by using blocking agents, which suppress the growth of ALD‐layers. As blocking agents, different kinds of polymers or self‐assembled monolayers are used. After the ASD process some blocking agent must be removed, which could be challenging, since it could affect the coating process.

As this work focuses on the processes of ASD for use on active implants, framework conditions are to be created, and only certain processes are to be used for closer evaluation:

1. Only a certain selection of materials can be considered for implant technology. For example, the neural implants consist of a polymer such as polyimide, Parylene‐C or PDMS, which serve as the substrate material,^[^ ^64^ ^]^ and a selection of noble metals, which are used as the electrode material.^[^ ^65^ ^]^ Furthermore, different kinds of metal oxides are used for encapsulation of the polymer substrates. These materials are therefore of particular interest for ASD.
2. The degree of selectivity is also an important criterion. When encapsulating neural implants using metal oxides, the encapsulation must reach a minimum thickness^[^ ^66^ ^]^ so that the barrier effect is sufficiently high before the selectivity window is exhausted and growth begins on the non‐growth areas.
3. Process control also plays an important role. High temperatures above 350 °C would damage the polymers used. Lower coating temperatures also ensure a lower thermal load due to thermally induced expansion and are therefore favorable.
4. To keep an eye on costs, susceptibility to errors and process time as low as possible, the ASD should be chemically self‐organizing. For example, processes in which polymers such as polymethylmethacrylate (PMMA),^[^ ^67^ ^]^ polyvinylpyrrolidone (PVP)^[^ ^68^ ^]^ or others are applied by spin coating for subsequent lithography processes, as well direct write methods, for area activation/passivation by means of microcontact printing,^[^ ^69^ ^]^ µ‐plasma pre‐treatment,^[^ ^70^ ^]^ e‐beam deposition^[^ ^71^, ^72^ ^]^ and scanning probe microscopy lithography^[^ ^73^ ^]^ are not considered. Only the fundamental subsequent surface reactions for achieving ASD are considered.
5. Finally, ASD process should be able to be integrated into existing processes for the coating and manufacturing of AIMDs.

#### 3.1.1 Inherent ASD for Active Implants

The nucleation delay of ALD layers depends on the surfaces to be coated, and the precursors being used. The difference is greater when two chemically different surfaces are involved.^[^ ^74^ ^]^ In the work by Cao et al.^[^ ^75^ ^]^ a wide spectra of the inherent selective ALD is discussed. Using inherent ASD, selective growth can be achieved not only on chemically different surfaces, but also on the same chemical surface. In addition to lattice defects, a real surface also contains imperfections, cracks and grain boundaries that can be exploited for nucleation. In 2D materials such as graphene, these defects can be used to enable the selective deposition of ALD, whereas on pristine graphene no ALD growth occurs.^[^ ^76^ ^]^ Yan et al.^[^ ^77^ ^]^ investigated a Pd_1_/graphene catalyst to selectively hydrogenate 1,3 butadiene. It would be conceivable to utilize this effect to selectively detect biomolecules for novelle approaches. Graphene itself supplies a solid base for building electrochemical sensors for the use in medical field.^[^ ^78^ ^]^ With the help of ASD, various biosensors for a wide range of biomolecules could be produced on carbon‐based platform^[^ ^79^ ^]^ or it can be used for selective coating of an induction layer for metal organic framework (MOF) particles.^[^ ^80^ ^]^ Kim et al.^[^ ^81^ ^]^ investigated a low‐temperature (165 °C) ALD process of platinum on graphene. Graphene has also potential to be used as a transparent conductive electrode material but has a high sheet resistance. The platinum grew selectively on defects of the graphene and reduced the sheet resistance while ensuring high transmission.

Another option for inherent selectivity is coating on step edges. This method is mainly used on nanoparticles for catalysts to make them more resistant, more activated and more stable.^[^ ^82^ ^]^

#### 3.1.2 Activated ASD for Active Implants

Many approaches that achieve ASD via surface activation use direct‐write or microcontact printing techniques. However, these steps would not be necessary for application to neural interfaces, as the existing product already has exposed noble metal surfaces. This means that work such as an e‐beam method for depositing platinum^[^ ^83^ ^]^ can be considered for activated ASD, as the focus here is on the fundamental reaction process of the noble metal surface. Activated ASD utilizes the high surface energies of noble metals such as Pt,^[^ ^56^, ^72^ ^]^ Pd,^[^ ^84^ ^]^ Au,^[^ ^85^ ^]^ or Ru.^[^ ^86^ ^]^ For example, oxygen is dissociative chemisorbed at the Pt surface. This allows a reaction to take place at these active sites in the subsequent precursor reaction step, leading to activated ASD. It is used to generate protective and functional overcoating and to produce core‐shell catalysts, which show improved selectivity, stability and activity.^[^ ^87^ ^]^

A connection between biological tissue and the electronics of the implant is established via the electrode surface. These electrodes are made of a noble metal such as Ptor Au. An activated ASD process could be used to make these electrode surfaces more efficient and resistant. The electrochemical reactions on the electrode surface cause the electrode to weaken.^[^ ^88^ ^]^ By using an activated ASD process, as used for nano shell particles in catalysts, the electrode surface could be similarly enhanced and made more resistant. To achieve a better charge‐injection, iridium oxide is sputtered onto the platinum electrodes.^[^ ^89^ ^]^ This process requires additional lithography steps, which is associated with time and costs. The iridium oxide could be applied area selectively to the Pt surface, saving time and costs.

In addition to activated ASD to improve performance, passivated ASD should also be considered, which is ideally suited to produce selective encapsulation.

#### 3.1.3 Passivated ASD for Active Implants

In addition to activating the surface to initiate ALD growth, it is also possible to passivate the surface with suitable species. For this purpose, polymers or self‐assembled monolayers are used. In almost all processes, passivation can only withstand a certain number of ASD cycles until failure. A practical example of surface passivation would be the encapsulation of neural implants. The encapsulation materials used include aluminum oxide, titanium oxide, hafnium oxide and silicon oxide. By passivating the device surface, the implant itself, including the side walls of the electrode could be encapsulated by a hermetic, electrically insulating, thin, and flexible coating, without impairing the electrode surface.

##### Self‐Assembled Monolayers (SAM)

**Figure 5:**
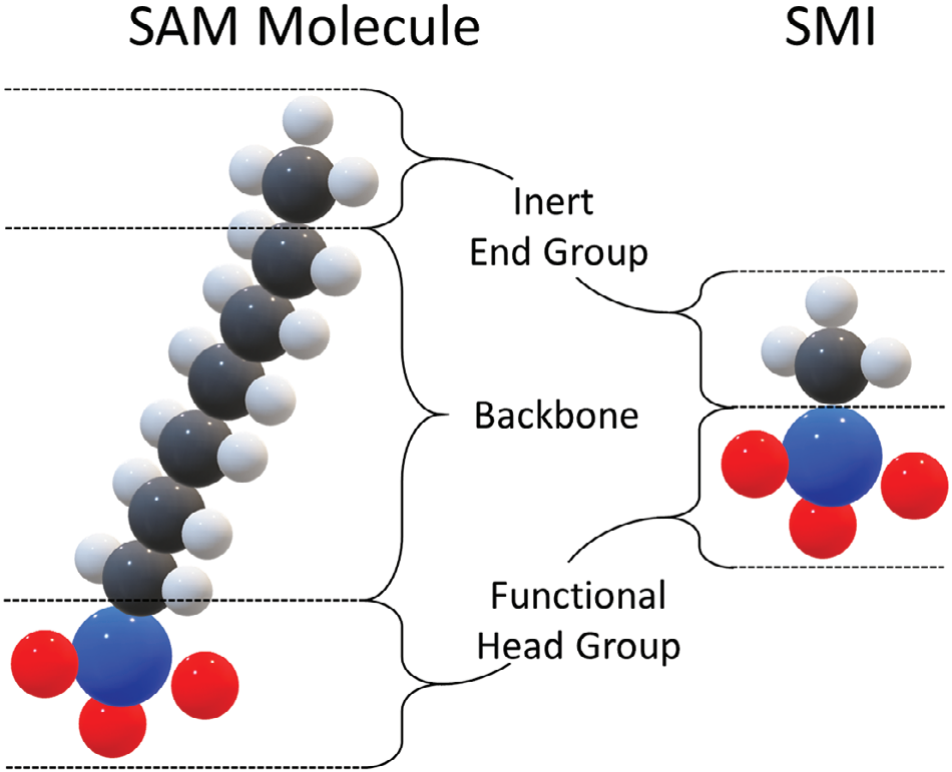
Comparison between SAM molecule and an SMI. The SMI has no backbone. Adapted with permission^[^ ^97^ ^]^ 2021, Journal of Vacuum Science & Technology A.

Self‐assembled monolayers are long‐chain molecules consisting of a body, a functional end group and a head group (**Figure** **5**). They were first studied in the 1940s by Zisman et al.^[^ ^90^ ^]^ SAMs adsorbs spontaneously on the surface and passivate it. To achieve passivation, alkyl end groups such as CH_3_ are used to prevent chemisorption of the precursor.^[^ ^91^ ^]^ Furthermore, longer chain molecules due to van der Waals forces provide a better organized structure, whereby the SAMs are densely packed and saturate the surface with fewer defects, leading to better passivation abilities.^[^ ^92^ ^]^ Appropriate head groups are selected so that the SAMs adsorb chemoselectively onto the surfaces to be passivated. For instance, to passivate metals like Au,^[^ ^93^ ^]^ Pd^[^ ^94^ ^]^ or Pt surfaces,^[^ ^95^ ^]^ alkylthios are used. Passivation of polydimethylsiloxane is done using appropriate silane head groups. They bind on the hydroxyl‐terminated surface, forming a Si‐O‐Si network. The OH groups need to be densely packed on the surface to prevent defects in the SAM layer, which could serve as nucleation sites for the ALD.

The SAMs can be applied either from a liquid phase or from the vapor phase. In the liquid phase, the SAMs are dissolved in an organic solvent such as toluene or hexane and the substrate to be passivated is placed in the solution for several hours or even days, which could alter the active implant through absorption and swelling. To prevent this problem from occurring, the SAMs should be applied to the substrate from the vapor phase. Ideally, the substrate is in the reactor where the ASD is to be carried out later, to prevent atmospheric contamination. Before the coating process, the SAMs are then introduced into the chamber in a similar way to the precursors, where they chemoselectively bind to the surface to passivate it.

Seo et al.^[^ ^96^ ^]^ showed an ASD of aluminum oxide on titanium in which certain areas were masked with octadecyl phosphonic acid (ODPA). The coating was carried out at 150 °C with TMA and water. By adapting the process, whereby the partial pressure of the TMA played an important role, it was possible to selectively deposit 60 nm of aluminum oxide. With a low partial pressure of the TMA of 1.3 Torr, the physisorption was greatly reduced and with a high argon purge pressure of 8.9 Torr, physiosorbed TMA could be purged away from the SAMs. It did not matter whether the purging step lasted 10 or 60 s. After the coating process, the ODPA was removed with oxygen plasma at 300 watts and 60 s.

However, the use of SAMs is limited, because the chemistry of the ALD process alters the SAMs and the smallest defects in the SAMs surface are sufficient to initiate ALD growth. To overcome this disadvantage, smaller, highly volatile inhibitors are used that function similarly to the SAMs.

##### Small Molecule Inhibitors

The small molecule inhibitors (SMI) are similar in structure to the SAMs and have an inert end group and a functional head group. The main difference is the lack of a backbone (Figure 5). SAMs and SMIs come with different properties (**Table** **1**). SAMs often cannot be applied from the vapor phase due to their size as they do not have good volatility. SMIs, on the other hand, have a high volatility, which not only allows them to be deposited from the vapor phase, but also makes them attractive for integration into existing ALD processes to generate an ASD process. If the passivating effect of the SMI decreases, they can be renewed directly from the vapor phase without vacuum breakage, which increases selectivity. However, they only have limited intermolecular forces due to the lack of a backbone, which means that the passivation quality is weaker than that of SAMs.^[^ ^97^ ^]^ Furthermore, SMIs can also serve as nucleation sites if they are not completely removed from the surface. Merkx et al.^[^ ^98^ ^]^ showed in the study of acetylacetone (Hacac) as SMI that the oxygen plasma process to remove the SMI, left behind fragments such as formates and carbonates, which acted as nucleation sites on the non‐growth area and thus led to a loss of selectivity. Merkx et al.^[^ ^99^ ^]^ used the aromatic inhibitor aniline to generate an ASD process for the selective deposition of titanium nitride on aluminum oxide and silicon oxide at 250 °C. Ruthenium (Ru) and cobalt (Co) were passivated with aniline. Aniline was integrated into an existing ALD process. The normal AB process was extended to an ABCD process. The inhibitor was introduced in step A and a bias voltage was applied in step D so that the inhibitor and impurities formed in Step C were removed by plasma. Layers of 6 nm TiN could be produced on the metal oxides until the selectivity decreased.

**Table 1:** Advantages (+) and Disadvantages (‐) of SAMs and SMIs.

+	SAM	−
High passivation potential due to highly ordered and dense packed structure		Unsuitable for sub 10 nm production Low volatility => Vapor application limited Passivation potential limited ALD chemistry alters SAMs

+	SMI	−
High volatility => Vapor application possible In vacuo regeneration of passivation layer possible Integration in existing ALD Process		Lower passivation potential since no ordered structure is formed Precursor could react with SMI itself or nearby reactive side

Khan et al.^[^ ^100^ ^]^ have used Bis(N,N dimethyl amino) dimethyl silane (DMADMS) and (N,N diethylamino) trimethyl silane (DMATMS), which are normally used as precursors for the ALD of SiO_2_. The SMI could be easily integrated into an ALD process to passivate an hydroxyl‐terminated surface to block the growth of Ru, Pt, Al_2_O_3_ and HfO_2_.

##### Topographic Passivation

Kim et al.^[^ ^101^ ^]^ investigated the implementation of topographic passivation. CF_x_ species were implanted using an acceleration voltage of 1 kV and a dose of 1 × 10^16^ ions cm^−2^ onto silicon fin arrays. The surfaces perpendicular to the implantation direction were provided with an ultra‐thin, hydrophobic layer. Selective coating of 500 Pt‐ALD cycles at 250 °C was possible, which results in a layer thickness of roughly 25 nm. This method could be used to increase the performance of the electrode of a neural implant, as the electrode surface could be enlarged. Ion implantation ensures that no ALD growth takes place on the surfaces of the entire implant that are perpendicular to the direction of implantation. The side walls would not be affected. As a result, a Pt layer could grow on the side walls, which would form an electrical connection with the electrode surface, thus increasing the geometrical surface.

#### 3.1.4 Tailored Process for ASD for Active Implants

In addition to passivating the surface or utilizing catalytic properties, selective deposition can also be made possible by adapting process parameters. For example, Weber et al.^[^ ^22^ ^]^ investigated the selective deposition of platinum from the precursor methylcyclopentadienyl(trimethyl)platinum (MeCpPtMe_3_) and oxygen as a reactant on previously deposited palladium nanoparticles on aluminum oxide. The process was carried out at 300 °C. They reduced the oxygen partial pressure to 7.5 mTorr during the reactant step. This made it possible to achieve a selectivity between palladium and aluminum oxide that withstood over 1000 coating cycles. The selective deposition is based on the dissociative chemisorption of oxygen on the catalytic platinum group metals and can be considered as activated ASD. Furthermore, they carried out another process to investigate the selective coating of palladium on platinum. They carried out the Pt‐ALD process at 300 °C at an oxygen partial pressure of 750 mTorr and were able to detect Pt‐nanoparticles after just 5 cycles on the aluminum oxide, proving that the partial pressures play an important role in selectivity. Subsequently, the palladium was selectively deposited on the platinum nanoparticles at 100 °C without vacuum breakage and hydrogen as reactant. No information regarding hydrogen partial pressure is given. Due to the catalytic effect of the platinum metals, the hydrogen molecules were dissociative chemisorbed, allowing the palladium layer to grow on the platinum and not on the aluminum oxide.

Additionally, Mackus et al.^[^ ^102^ ^]^ investigated the total exposure of the oxygen at the same conditions, by varying the pulse time and pressure. Longer pulse times have the same effect on nucleation as an increased pressure. Leading to the conclusion, that the product of time and pressure is the parameter for the nucleation delay. This is of particular importance for coating reactors that do not allow a higher coating pressure due to their design. The pulse time can be used to counteract this.

The reactant itself also has an influence on selectivity. For example, the deposition of platinum on graphite, in which oxygen was used as a reactant, resulted in selective deposition at the step edges of the graphite. When ozone was used instead of oxygen, the entire graphite was coated with platinum. The ozone etched the graphite and thus created new step edges on which the platinum precursor could nucleate.^[^ ^103^ ^]^

In addition to the use of the reactant, the choice of precursor also plays an important role. In their work, Oh et al.^[^ ^104^ ^]^ investigated various designs of an aluminum precursor (Al(CH_3_)_x_Cl_3−x_ (x = 0, 2, and 3) and AlC_y_H_2y+1_ (y = 1 and 2)) and the effects on an ASD process using Octadecyl trichlorosilane (ODTS) as the SAM. Aluminum oxide provides outstanding barrier properties and is biocompatible. The coating was carried out at 200 °C on silicon surface and SAM‐terminated silicon surface using H_2_O as the reactant. By increasing the purging time, the content of aluminum on the blocking SAMs decreased, enabling better selectivity. The chlorine precursors tend to dimerize, creating larger molecules that can be more easily purged from the SAM surface. On the other hand, Al(CH_3_)_3_ is a small molecule and a strong Lewis acid, which tends to favorably dimerize at 200 °C. As a result, it easily adsorbs and absorbs into the SAM surface, making a selective coating process highly challenging. The best selectivity of >0.98 was achieved, using Al(C_2_H_5_), which corresponds to a film thickness of 6 nm. Sinha et al.^[^ ^105^ ^]^ investigated the influence of the precursor chemistry of precursors on the ASD of titanium oxide. Titanium isopropoxide (TTIP, Ti [OCH(CH_3_)_2_]_4_) and titanium tetrachloride (TiCl_4_) were used for the experiments. PMMA was used as the blocking agent. The coating was carried out on silicon at 160 °C with water as the reactant. The organometallic precursor TTIP was much easier to block than the TiCl_4_ precursor. TiCl_4_ is a strong Lewis acid, and they hypothesized that a coordinative bonding occurred between the precursor and the C═O group of PMMA, which degrades the selectivity. TTIP is a relatively large molecule, which worsens the diffusion through the PMMA. Its reactivity with PMMA is also very low, which is why it can be blocked over 500 cycles. This shows that the choice of precursors also has a major influence on the feasibility of ASD. A theoretical evaluation of design strategies was done by Kim et al.^[^ ^106^ ^]^

Many of the processes presented often have limited selectivity. To increase this, so‐called super cycles can be incorporated into the ASD process. Here, an etching step is inserted after a certain number of ASD cycles, which removes the material on the non‐growth area and thus improves selectivity. A directed plasma process or atomic layer etching (ALE) can be used for removal of residues on the non‐growth area. In their work, Song et al.^[^ ^107^ ^]^ showed the improvement of the selectivity of a titanium oxide process (TiCl_4_ as the precursor and deionized water as reactant) on a Si‐OH surface (growth area) against a Si‐H surface (non‐growth area) to over 12 nm by using 5 cycles of ALE after 30 cycles of ASD at a temperature of 170 °C. ALE was carried out using wolfram hexafluoride (WF_6_) and boron trichloride (BCl_3_). V*os* et al.^[^ ^108^ ^]^ demonstrated another super cycle process by removing deposited ruthenium on a silicon oxide and an aluminum oxide surface with oxygen plasma. The etching was done using a plasma power of 100 W. The focus of this work was to minimize the trade‐off between selectivity and net‐deposition, since material is also removed from the growth area during etching process. They used a process temperature of 150 °C whereas the difference between growth per cycle was biggest between the non‐growth area and growth area and varied the duration and etch frequency of the oxygen plasma.

### 3.2 Challenges Using Area‐Selective Atomic Layer Deposition

#### 3.2.1 Loss of Biocompatibility

One of the most important aspects of active implants is their biocompatibility and their effect on the human organism. The international standard ISO 10993^[^ ^109^ ^]^ defines measures to perform a comprehensive biological evaluation of medical devices. The deposited layers must also comply with the requirements of the standard with respect to toxicity and sensitization. In addition, REACH^[^ ^110^ ^]^ and RoHS^[^ ^111^ ^]^ need to be considered as they ban certain substances in general use. It can therefore be difficult to generate an ASD process for use on active implants, as this is subject to strict regulations in the AIMD area. The biocompatibility of titanium oxide,^[^ ^45^, ^112^ ^]^ hafnium oxide,^[^ ^46^ ^]^ and aluminum oxides^[^ ^113^ ^]^ produced using ALD has already been demonstrated. However, changing the processes to generate an ASD process can mean a loss of biocompatibility. Even the smallest impurities in the layer could lead to allergic reactions or even cell death. Due to current standards, the choice of chemicals for the ASD process is also very limited. For example, topographic ASD using fluoropolymers would no longer be possible due to the PFAS^[^ ^114^ ^]^ regulation. The use of SAMs or SMIs could also lead to contamination of the layers, which would impair biocompatibility. The reactor used must also be approved for use on medical devices, which can also be an obstacle.

#### 3.2.2 Adhesion Strength

Another challenge is the adhesion strength of the functional layers to the electrode material and the polymers of the implant. A functional layer is only as good as its adhesion to the substrate material. Barrier layers cannot adhere with high binding energy to the polymers, since polymers are often non‐polar and therefore offer no chemical bonding partners for precursors. This can be counteracted by using proper plasma pretreatment.^[^ ^115^ ^]^ Similarly, some polymers used as substrate materials in implant technology are also used as passivation layers in ASD, such as polyimides.^[^ ^116^ ^]^ This shows that the coating of the implants goes against the “chemical nature” of the surfaces. This requires a careful selection of pretreatment steps and process parameters to ensure a successful ASD process with sufficient adhesion.

#### 3.2.3 Influence of Process Technology on Neural Implants

The processes used to perform the ASD require pre‐treatment and the coating process itself can change the properties of the implants. For example, an organic solvent is used for the application of SAMs, which can destroy the polymers of the substrate due to swelling. The high process temperatures during coating improves the film qualities, with higher densities and lower impurities,^[^ ^117^ ^]^ but can also alter the polymers. The glass transition temperature and the coefficient of thermal expansion should be considered, when coating polymers, since they can have a negative effect on the barrier properties.^[^ ^118^ ^]^ The different coefficient of thermal expansion between the coating and polymer can lead to stresses during the heating and cooling process, which can promote detachment.^[^ ^119^ ^]^ The precursors used can also change the substrate material.^[^ ^120^ ^]^ For example, it has been observed that TMA can react under the polymer surface and thus become incorporated into the backbone of the polymer.^[^ ^121^ ^]^

#### 3.2.4 Non‐Sufficient Selectivity

The barrier layers must be sufficiently thick to ensure adequate barrier protection of the implants. An ASD process with which <10 nm aluminum oxide can be deposited would not be sufficient for use on active implants, since the barrier properties would not be sufficient.^[^ ^122^ ^]^ For the semiconductor industry, a layer thickness of just a few nanometers would be sufficient for gate oxides, but for active implants the layer must be thick enough to have outstanding barrier properties. To achieve the best barrier layers, several different metal oxides are deposited one after the other to create a layer system that has the best barrier properties.^[^ ^123^ ^]^ This also leads to further challenges as the correct parameters and chemicals must be used for each layer system to enable selective growth.

## 4 Conclusion

Area selective atomic layer deposition would be the technology of choice to achieve hermetic protection of sensitive electronic circuits in chip‐in‐foil systems and increase electrochemical surface area on recording and stimulation electrodes for flexible and miniaturized neural implants. While the processes as such are established, transfer into the field of active implantable medical devices poses still challenges with respect to materials, process residues and validation of implant longevity when the first systems would have been developed for chronic implants. Envisioning potential benefits of this approach, it seems to be worth to drive concerted actions to meet the challenges and introduce a new generation of neural implants.

## Conflict of Interest

The authors declare no conflict of interest.
